# DNA Watermarking of Infectious Agents: Progress and Prospects

**DOI:** 10.1371/journal.ppat.1000950

**Published:** 2010-06-17

**Authors:** Daniel C. Jupiter, Thomas A. Ficht, James Samuel, Qing-Ming Qin, Paul de Figueiredo

**Affiliations:** 1 Department of Surgery, Scott & White Memorial Clinic and Hospital, Temple, Texas, United States of America; 2 Department of Veterinary Pathobiology, Texas A&M University, College Station, Texas, United States of America; 3 Department of Microbial and Molecular Pathogenesis, Texas A&M Health Science Center College of Medicine, College Station, Texas, United States of America; 4 Department of Plant Pathology and Microbiology, Texas A&M University, College Station, Texas, United States of America; The Fox Chase Cancer Center, United States of America

Following the 2001 anthrax attacks, infectious disease research laboratories and personnel were subjected to increased scrutiny amid concerns that the released agent originated from within such facilities. Since then, enhanced regulatory controls have been implemented to thwart the possibility of future releases. However, improved microbial forensics technologies have not been employed to facilitate fault attribution or to control and track agent inventories.

We believe that novel systems employing enhanced identity protection will instill new public confidence in scientists and avoid erroneous assignment of liability in the case of a release. We propose a DNA watermarking system that includes institution-, laboratory-, and/or investigator-specific watermarks in the genomes of organisms, especially Select Agents. The system will achieve five key goals critical to any watermarking system, phrased in general information theoretic terms: message fidelity, error tolerance, ease of interpretation, availability of signatures, and resistance to attack (Table 1).

**Table 1:** Goals and features of watermarking system.

Key Goal	Feature Description	Feature Implementation	Feature Benefit	Example
Message fidelity	Watermark does not disturb phenotype of organism.	Watermarks integrated into neutral or selected loci.	No mutual interference between genomic and watermark signal.	Telephone conversations carried on one cable.
Error tolerance	Watermark is robust to insertion, deletion, and point mutations.	Watermarks designed for redundancy and encryption. Large pairwise distance between watermark sequences.	Recover identification in spite of damage to information.	JPEG compression algorithm.
Ease of interpretation by intended receiver	Watermark can readily be recovered by authorities.	Watermarks integrated into defined and stable loci.	Recoverable by appropriate entities.	Computer password encryption.
Availability of signatures	Each lab receives a unique signature.	Suitably long watermark sequences. Large pairwise distance between watermark sequences.	Nearly unlimited signatures.	Credit card numbers.
Resistance to malicious attack	Abundance of watermark sequences prevents fabrication of authentic sequences.	Suitably long watermark sequences. Large pairwise distance between watermark sequences.	Signature complexity and length provide security.	Credit card numbers.

**Figure 1:**
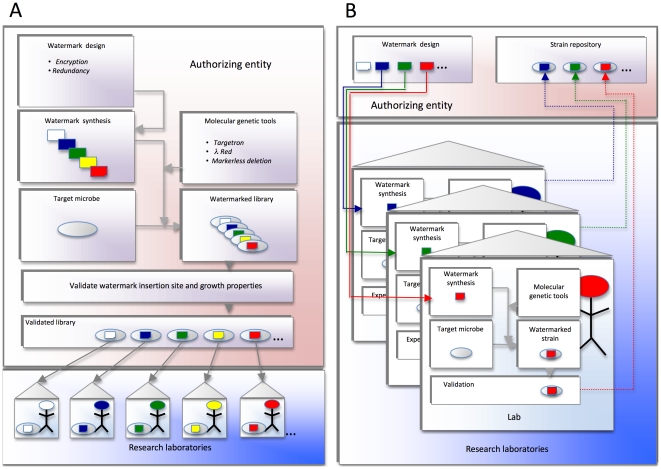
Proposed watermark implementation strategies. (A) Schema in which a central authorizing entity distributes watermarked strains to the research community. The authorizing entity designs and synthesizes confidential DNA watermark sequences. These sequences are introduced into genetically tractable target organisms using molecular genetic approaches that have proven efficacious in the agent. Proper insertion of watermarks at the desired loci is confirmed. The growth properties are also validated. The watermarked strains are finally distributed to individual labs, institutions, or investigators for experimental use. (B) Schema in which a central authorizing entity distributes to the research community DNA sequences that can be used for watermarking strains. The authorizing entity designs and distributes confidential DNA sequences for watermarking strains (solid lines). Individual institutions, laboratories, or investigators use this DNA to watermark their experimental strains. The individual research entities also confirm the proper integration of watermarked DNA sequences into the appropriate target genomic loci, and characterize the phenotypes of the resultant watermarked strains. The authorizing entity can also serve as a repository for watermarked strains (dotted lines).

A DNA watermark is a unique synthetic DNA sequence embedded into the genome of a genetically tractable organism. The watermark provides a means for agent, isolate, or strain identification and tracking by PCR amplification and sequencing of the embedded tag. The power of watermarking for agent control emerges when the technology is linked to the activities of a trusted authorizing entity (Figure 1). This entity could, for example, be charged with distributing organisms containing unique watermark sequences to individual laboratories and/or investigators. These watermarks would distinguish their organisms from those of others in the research community. Laboratories would be encouraged, permitted, or required to use only strains that contain their approved, and confidential, watermark. In the event of release, the offending pathogen would be interrogated for the presence of an approved watermark. If such a watermark were present, then information about the possible source would become immediately available. Of course, pathogen-specific standard operating procedures (SOPs) that ensure the integrity of the watermarking system (to prevent cross-contamination, manage the sharing of strains, and prevent accidental or intentional misuse) would be a necessary component of any watermarking strategy.

Previously developed watermarking technologies include approaches for embedding watermarks in microbial genomes [1]–[4] and strategies for encryption [2], [5], [6]. Each method seeks to develop a genetic cipher that is 1) robust to mutation, 2) easy for intended users to decipher, and 3) difficult for third parties to decipher or alter. While these strategies for manipulating watermarks have been successful at watermark encoding, placement in a genome, retrieval from a genome, and decoding, none of the techniques achieves all of the five goals (outlined in Table 1) that are necessary for a watermarking system for Select Agent tracking.

In our opinion, however, these techniques are worthy of further investigation, as regards their utility for the research and biosecurity communities. We propose investigation proceed on three discrete but interconnected fronts. First, the theoretical mathematical and information aspects of watermarking systems must be examined and rigorously tested in silico. Second, insertion and removal of watermarks from microbial genomes must be assessed, and the phenotypic invisibility of the watermarks tested. Finally, pathogen-specific SOPs must be developed, keeping in mind the need for transparency and collaboration in research, and tested in a “role playing” scenario. Our initial work indicates that the model is mathematically plausible. Previous work in the use of watermarks suggests that appropriately placed watermarks can be phenotypically neutral [1], [3]. The technologies to introduce watermarks into several of the highest risk Select Agent genomes are currently available, using site-specific insertion tools such as Targetron (intron-based homing) and Lambda red mutagenesis. Adaptation of these or comparable genetic tools for less tractable Select Agents would require technological advances that would also broadly benefit research of each agent.

Adoption of a watermarking strategy by research groups would need to be justified by a cost-benefit analysis, from an institutional liability perspective, and from the perspective of the research community. Several salient concerns can be readily identified. Our proposed system does not protect against covert usage of naturally occurring wild-type strains or remediate existing stocks, but instead provides a forward-looking strategy. To address cost concerns, funding agencies that require enhanced inventory control could be encouraged or required to support the cost of implementing watermarking systems. Similarly, these agencies could support or collaborate with private or public authorizing entities to develop SOPs for strain management. Finally, convincingly establishing phenotypic neutrality of genomic modifications will be non-trivial, and thus, will constitute an important area for future research. Despite these potential impediments, watermarking would nearly eliminate the potential for mistaken assignment of source for a suspected agent release. Moreover, the development and implementation costs may prove to be much less than other proposed measures for enhancing laboratory security, including around-the-clock security patrols.

We considered two variations on the operational infrastructure required (Figure 1). An authorizing entity could (Figure 1A) design, insert, and distribute, or (Figure 1B) simply distribute, the secured watermark to requesting laboratory. In the latter scenario, the requesting laboratory would be responsible for adapting genetic technology to deliver the watermark. We do not propose that previously generated modified strains (mutant collections, etc.) would be modified and restocked. The transition to marked strains would be incremental but stable. We speculate that an efficient approach to this scenario would be to provide funding opportunities to establish and validate agent-specific systems. While there are several potential impediments to implementing the proposed watermarking systems, the combination of positive impact on lay perception of responsible scientific activity and an increased confidence in control of liability by investigators and institutions provides a rationale to investigate the development of watermarking tools for Select Agent research.
